# Sexual behavior and associated factors in rural adolescents

**DOI:** 10.11606/S1518-8787.2018052006988

**Published:** 2018-04-04

**Authors:** Bárbara Cabral de Sousa, Rebeca Silva dos Santos, Katiuscy Carneiro Santana, Raquel Souzas, Álvaro Jorge Madeiro Leite, Danielle Souto de Medeiros

**Affiliations:** I Universidade Federal da Bahia. Núcleo de Epidemiologia e Saúde Coletiva. Instituto Multidisciplinar de Saúde. Vitória da Conquista, BA, Brasil; II Escola Nacional de Saúde Pública Sérgio Arouca. Residência Multiprofissional em Saúde da Família. Rio de Janeiro, RJ, Brasil; III Prefeitura Municipal de Vitória da Conquista. Secretaria Municipal de Saúde. Vitória da Conquista, BA, Brasil; IV Universidade Federal do Ceará. Faculdade de Medicina. Departamento de Saúde Comunitária. Fortaleza, CE, Brasil

**Keywords:** Adolescents, Sexuality, Adolescent Health, Rural Areas, African Continental Ancestry Group, Adolescente, Sexualidade, Saúde do Adolescente, Zona Rural, Grupo com Ancestrais do Continente Africano

## Abstract

**OBJECTIVE:**

To describe the sexual behavior and to identify associated factors in adolescents from rural communities in Bahia, Brazil.

**METHODS:**

This is a cross-sectional, population-based, and household-based study, carried out in 2015 with adolescents aged 10 to 19 years. We described the variables of sexual intercourse in life and in the last 12 months, age at first intercourse, condom use and number of partners, guidance on pregnancy, AIDS, or other sexually transmitted infections, and guidance on how to get condoms. The analysis was performed for the total sample and for the *quilombola* and *nonquilombola* strata. We used Poisson regression, with robust variance, to estimate the prevalence ratios for sexual intercourse in relation to the explanatory variables.

**RESULTS:**

A total of 390 adolescents were interviewed, of them 42.8% were *quilombolas*, 51.3% females, and the median age was 14.8 years. Of these adolescents, 26.4% reported sexual intercourse (28.1% *quilombolas* and 25.1% *non-quilombolas*), and the median age of the first relation was 15 years; 77.7% of them mentioned condom use in the last intercourse and more than half received guidance on pregnancy, AIDS, or other sexually transmitted infections and received no guidance on how to get free condoms. Age (PR = 1.42) and alcohol use experimentation (PR = 2.41) were positively associated with sexual intercourse after adjustment. In the *quilombola* stratum, age (RP = 1.37), having three or more close friends (PR = 1.37), and experimentation with alcohol (PR = 2.60) were associated. In the *non-quilombola* stratum, age (PR = 1.43), black persons (PR = 2.06), and alcohol use experimentation (PR = 2.68) were associated.

**CONCLUSIONS:**

Lack of information and exposure to behaviors such as alcohol use experimentation are conditions that need to be addressed in health promotion strategies and in strategies for the prevention of sexual health problems in rural adolescents. Intersectoral partnerships between education and health also need to be strengthened to promote the autonomous and safe exercise of sexuality in this population.

## RESUMO

**OBJETIVO:**

Descrever o comportamento sexual e identificar fatores associados em adolescentes de comunidades rurais da Bahia.

**MÉTODOS:**

Estudo transversal, de base populacional e abordagem domiciliar, realizado em 2015, com adolescentes entre 10 e 19 anos. Foram descritos: relação sexual alguma vez na vida e nos últimos 12 meses; idade na primeira relação sexual; uso de preservativo e número de parceiros; orientação sobre gravidez, aids ou outras infecções sexualmente transmissíveis e como conseguir preservativo. Foi realizada análise para a amostra total e para os estratos quilombola e não quilombola. Utilizou-se regressão de Poisson, com variância robusta, para estimar as razões de prevalências para a relação sexual em relação às variáveis explicativas.

**RESULTADOS:**

Foram entrevistados 390 adolescentes, sendo 42,8% quilombolas, 51,3% do sexo feminino e mediana de idade de 14,8 anos. Desses, 26,4% relataram ocorrência de relação sexual (28,1% quilombolas e 25,1% não quilombolas), com mediana de idade da primeira relação com 15,0 anos; 77,7% mencionaram o uso de preservativo na última relação e mais da metade recebeu orientações sobre gravidez, aids ou outras infecções sexualmente transmissíveis e não recebeu orientações sobre como conseguir preservativo gratuito. Mostraram-se positivamente associados à ocorrência de relação sexual, após ajuste: idade (RP = 1,42) e ter experimentado uma dose de álcool (RP = 2,41). No estrato quilombola, a idade (RP = 1,37), ter três e mais amigos próximos (RP = 1,37) e experimentação de álcool (RP = 2,60) mostraram-se associados. No não quilombola, mostraram-se associados idade (RP = 1,43), raça/cor negra (RP = 2,06) e experimentação de álcool (RP = 2,68).

**CONCLUSÕES:**

A carência de informações e a exposição a comportamentos como o uso de álcool são condições que necessitam ser contempladas nas estratégias de promoção de saúde e prevenção de agravos à saúde sexual de adolescentes de zona rural. Ainda há necessidade de potencializar parcerias intersetoriais entre educação e saúde para fomentar o exercício autônomo e seguro da sexualidade nessa população.

## INTRODUCTION

In times of demographic transition and consequent population aging, 17.9% of the Brazilian population is made up of adolescents (10–19 years)^a^. Of these adolescents, 18% live in rural areas, where social inequities from the insufficient guarantee of citizenship rights are accentuated^a^ ^,^ ^b^.

As a process, adolescence circumscribes a series of “(…) transitions that form a cycle of life”^1^. Physiologically, this phase of human development begins with the first signs of puberty^c^. The transition to adulthood happens among the maturation of organs and systems, the personal trajectory, and the experiences of family and social contexts^1^. Curiosity guides discoveries and experiments and there is a distancing from the parental figures and greater appreciation of the peer group toward the emancipation and socialization of adolescents^1^.

Among the “first-time experiences” that occur in adolescence, the first sexual intercourse can be considered the rite of greatest repercussion in this phase^2^. There is influence of socioeconomic, cultural, and gender factors and, in the rural area, there is also a tendency to preserve more conservative behaviors in relation to gender relations and sexual behavior^3^.

In these rural communities, the geographic dispersion, the difficulty of access, and the limitations in the quality of health services show a greater precariousness when compared to the urban health conditions^b^. Adolescents are sometimes deprived of access to formal education, health services, and leisure and work opportunities, which are of great importance for their development^c^.

In this scenario, the research named ADOLESCER emerged as a possibility to carry out a comprehensive diagnosis of the health conditions of rural adolescents. It sought to respond to a demand that primary care professionals had to know the epidemiological profile, understand its health-disease-care process, and problematize care strategies for this population segment that is still distant from health services. The municipality of Vitória da Conquista, the research headquarters, is located in the southwestern region of Bahia, Brazil, and has a population of 306.806 inhabitants^a^, of which 11.7% live in an extensive rural area, also made up of *quilombola* communities.

Considering the exposed situation and the lack of scientific productions related to the sexual health of rural adolescents in Brazil, this research was guided by two questions: “What is the sexual behavior of the adolescents of a rural region in Bahia?” and “Are there related individual and social vulnerabilities?”.

Based on these questions, we defined as objective the description of the sexual behavior of rural adolescents of a municipality in Bahia and the identification of individual factors and the associated family and social contexts.

## METHODS

This is an analysis of the results of the research named “*ADOLESCER: Saúde do Adolescente da Zona Rural e seus condicionantes*”, carried out in rural communities within the State of Bahia, Brazil. It was a cross-sectional, population-based, and household-based study that used a structured questionnaire.

The research project was approved (Process 57384) by the Research Ethics Committee of Universidade Federal da Bahia – Campus Anísio Teixeira. All adolescents aged 18 years or more signed the Informed Consent (IC). Adolescents younger than 18 years signed the informed consent, expressing their agreement to participate in this study, only after their guardians signed the IC.

To ensure the representativeness and viability of the research, we chose a sampling strategy that considered the territorial extension and the population of adolescents living in rural communities. Thus, we used as sample principles the selection of proportional households for the number of adolescents per community and the interview of only one adolescent per household. In addition, the sample was calculated separately for each stratum so we could obtain valid estimates for the *quilombola* and *non-quilombola* populations.

In order to carry out the population estimate, we used the data of Record A completed by the community agents during the household visits. At the time of the research, Vitória da Conquista had coverage of 97.4% of the Program of Community Health Agents in the rural area. The sample universe consisted of 811 adolescents, divided into the strata *quilombolas* (n = 350), residents of *quilombola* communities recognized by the *Fundação Palmares*, and *non-quilombolas* (n = 461).

The sample calculation considered a prevalence of 50%, accuracy of 5%, 95% confidence level, design effect equal to 1.0%, and 15% for possible losses. However, considering that only one adolescent per household would be interviewed and the number of households for the *quilombola* group would be greater, all households in the *quilombola* communities were visited, resulting in an increase of 7.1% for losses in this stratum.

We excluded from the study adolescents or guardians who were unable to respond to the questionnaire because they were inebriated at the moment of data collection or presented severe mental disorders with cognitive impairment.

Sampling for *non-quilombola* adolescents occurred in two stages: 1) random selection of households with adolescents, according to the proportional distribution of adolescents per community, 2) random selection of adolescents in each household. In the *quilombola* households, we only randomly selected the adolescents in the household.

The data collection instrument, structured in two sections, was elaborated from surveys^1^ ^,^ ^d^ ^,^ ^e^ carried out in Brazil. The first one, to be answered by the guardian of the adolescent or the resident aged 18 years or more, investigated the general characteristics of the household, income, and education of the head of the family. The second section, answered by the adolescent, investigated the characteristics of the adolescent and social support, job, lifestyle, perception of health status and body image, use of illicit drugs, sexual and reproductive health, and use of health services. A pilot study was conducted in December 2014 in a rural community not participating in the main study.

Before collecting the data, we presented the research at the *Quilombola* Local Health and Territorial Councils and at meetings of the Family Health Strategy teams, for support and dissemination to the community. Household sensitization occurred simultaneously with mapping. For mapping, we used a GPS device, eTrex-30 (Garmin).

Interviewers were previously trained and they carried out the interviews using portable computers (HP Pocket Rx5710). Data collection occurred between January and May, 2015.

To describe the sexual behavior of adolescents, we used the following variables: sexual intercourse in life, age at first intercourse (in years), sexual intercourse in the last 12 months, condom use in the last intercourse, and number of sexual partners in life. To describe the guidance received by the adolescents, we used the following variables: guidance on pregnancy, AIDS, or other sexually transmitted infections (STI) and guidance on how to get condoms.

The dependent variable in this study was sexual intercourse in life, obtained from the question: “Have you ever had sexual intercourse (sex)?”. The independent variables were established from a review of the literature for the analysis of sexual behavior and associated factors, organized into three blocks (Figure).

**Figure:**
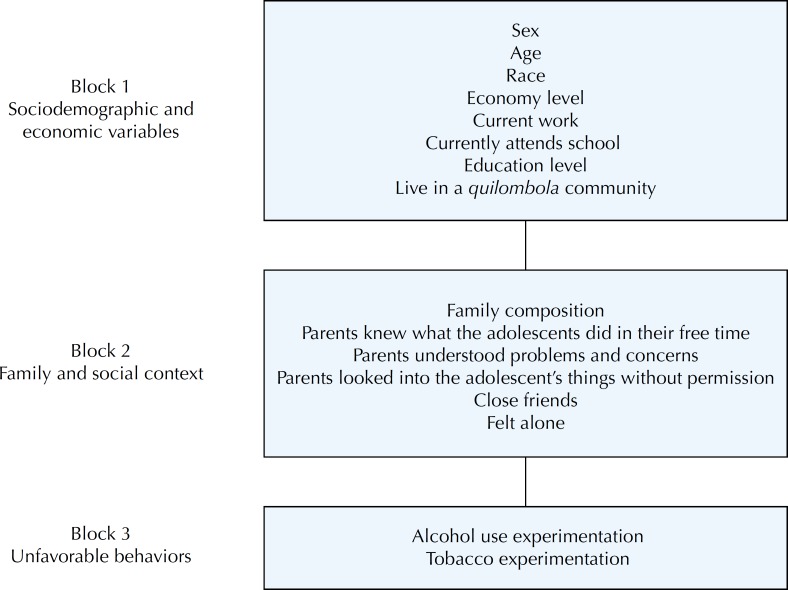
Theoretical model for the study of the sexual behavior of adolescents and associated factors. Research ADOLESCER, Bahia, Brazil, 2015.

The first group consisted of socio-demographic and economic variables: sex, age (in years), race, economic level (according to the criteria of the 2014 Brazilian Association of Research and Markets [ABEP]^f^), current work, currently attending school, education (in years of study), and living in a *quilombola* community.

In the second block, the family and social context was described based on the following variables: family composition, frequency with which parents knew what the adolescents did in their free time, frequency with which parents understood problems and concerns, frequency with which parents looked into the adolescent's things without permission, close friends, and feeling alone.

The third block, consisting of unfavorable behaviors, used the variables: alcohol use experimentation and tobacco experimentation.

The analyses were carried out for total sample and for each stratum, *quilombola* and *non-quilombola*. We carried out frequency distribution and dispersion analysis by median. Differences between proportions were tested with Pearson's chi-square or linear trend distribution or Fisher's exact test. The non-parametric variables were compared using the Mann-Whitney test. Prevalence ratio (PR) was used to estimate the association between the report of sexual intercourse and the explanatory variables of interest. Poisson regression with robust variance was used to estimate the PR for the report of sexual intercourse adjusted for potential confounding factors. We included all the variables in the initial model that showed association at a level of significance below 20% in the bivariate analysis. For all the tests and for the permanence of the variables in the final model, we used the significance level of 5%. The models were compared by the Akaike criterion. The adequacy of the model was assessed by the chi-square test.

To evaluate the effect of the losses on the outcome, we carried out a calibration of the natural expansion factors^4^. The estimates of sexual intercourse were compared using the chi-square test. We used the program Stata, version 12.0, for data analysis.

## RESULTS

We conducted 420 home interviews, 179 of which were in *quilombola* households (9.1% of losses) and 241 were in *non-quilombola* households (0.4% of losses). We interviewed 390 adolescents, being 167 *quilombolas* and 223 *non-quilombolas*, with losses of 15.2% and 7.9%, respectively.

The losses were differential in relation to sex, with a higher prevalence in males, for *non-quilombola* adolescents (p = 0.038). However, the estimate of the outcome, with and without the calibration factor for this variable, did not present significant differences in the estimates. In this way, we did not consider it in the analyses performed.

Sexual intercourse in life was reported by 26.4% of the adolescents (Table 1), and median age of the first intercourse was 15 years (minimum of six and maximum of 19 years). Of these adolescents, 74.8% had sexual intercourse in the 12 months before the interview, 77.7% mentioned condom use in the last relationship, and 35.9% had three or more sexual partners in their lives. Most adolescents received guidance on pregnancy (52.3%) and AIDS or other STI (56.1%) and received no guidance on how to get free condoms (57.7%) (Table 1).

**Table 1:** Description of the sexual behavior of and guidance received by adolescents from rural areas. Research ADOLESCER, Bahia, Brazil, 2015.

Variable		Total		*Quilombola*		*Non-quilombola*		p^b^
		n^a^	%	n^a^	%	n^a^	%	
Sexual intercourse in life								0.502
	Yes	103	26.4	47	28.1	56	25.1	
	No	287	73.6	120	71.9	167	74.9	
Sexual intercourse in the last 12 months								0.019
	Yes	77	74.8	30	63.8	47	83.9	
	No	26	25.2	17	36.2	9	16.1	
Condom use in the last sexual intercourse								0.897
	Yes	80	77.7	36	77.6	44	78.6	
	No	22	21.3	11	23.4	11	19.6	
	Refused to answer	1	1.0	0	0.0	1	1.8	
Number of sex partners in life								0.811
	1	43	41.8	19	40.4	24	42.9	
	2	19	18.4	10	21.3	9	16.1	
	≥ 3	37	35.9	17	36.2	20	35.7	
	Did not know/Refused to answer	4	3.9	1	2.1	3	5.4	
Guidance on pregnancy								< 0.001
	Yes	204	52.3	67	40.1	137	61.4	
	No	186	47.7	100	59.9	86	38.6	
Guidance on AIDS or other STI								0.001
	Yes	219	56.1	77	46.1	142	63.7	
	No	171	43.9	90	53.9	81	36.3	
Guidance on how to get free condoms								0.073
	Yes	165	42.3	62	37.1	103	46.2	
	No	225	57.7	105	62.9	120	53.8	

STI: sexually transmitted infection

a Absolute frequency.

b p value calculated by Pearson's chi-square or Fisher's exact test.

Among the *quilombola* adolescents, 28.1% reported sexual intercourse in life, while among the *non-quilombola* adolescents 25.1% reported it (Table 1), and the median age was 15 years in both strata (minimum and maximum of six and 19 years for the *quilombola* and 12 and 19 years for the *non-quilombola*, respectively). Significant differences were observed between the groups related to sexual intercourse in the last 12 months (p = 0.019), guidance on pregnancy (p < 0.001) and on AIDS or other STI (p = 0.001) (Table 1).

Most of the interviewees did not live in *quilombola* communities (57.2%), were female (51.3%), black (76.4%), belonged to economic level D or E (61.3%), did not work (61.8%), and attended school (91.0%) (Table 2). Median age was 14.8 years and education level was six complete years of study (minimum of one and maximum of 15 years).

**Table 2:** Description of the socio-demographic characteristics and behaviors of the study population. Research ADOLESCER, Bahia, Brazil, 2015.

Variable		Total		*Quilombola*		*Non-quilombola*		p^b^
		n^a^	%	n^a^	%	n^a^	%	
*Quilombola*								-
	No	223	57.2	-	-	-	-	
	Yes	167	42.8	-	-	-	-	
Sex								0.237
	Male	190	48.7	76	45.5	114	51.1	
	Female	200	51.3	91	54.5	109	48.9	
Race								0.003
	Non-black	92	23.6	27	16.2	65	29.2	
	Black	298	76.4	140	83.8	158	70.8	
Economy level								< 0.001
	B or C	151	38.7	40	23.9	111	49.8	
	D or E	239	61.3	127	76.1	112	50.2	
Current work								0.023
	No	241	61.8	114	68.3	127	56.9	
	Yes	149	38.2	53	31.7	96	43.1	
Currently attends school								0.281
	No	35	9.0	18	10.8	17	7.6	
	Yes	355	91.0	149	89.2	206	92.4	
Family composition								0.268
	Live with both parents	264	67.7	107	64.1	157	70.4	
	Only live with the father or mother	91	23.3	41	24.5	50	22.4	
	Do not live with parents	35	9.0	19	11.4	16	7.2	
Frequency in which parents knew what they did in their free time								0.559
	Most of the time/Always	239	62.6	98	59.8	141	64.7	
	Sometimes	70	18.3	31	18.9	39	17.9	
	Never/Rarely	73	19.1	35	21.3	38	17.4	
Frequency in which parents understood problems and concerns								0.984
	Most of the time/Always	163	42.3	70	42.2	93	42.5	
	Sometimes	124	32.2	53	31.9	71	31.4	
	Never/Rarely	98	25.5	43	25.9	55	25.1	
Frequency in which parents looked into their things without permission								0.131
	Most of the time/Always	53	13.8	19	11.5	34	15.5	
	Sometimes	58	15.1	20	12,0	38	17.4	
	Never/Rarely	274	71.1	127	76.5	147	67.1	
Close friends								0.731
	< 3	66	16.9	27	16.2	39	17.5	
	≥ 3	324	83.1	140	83.8	184	82.5	
Felt alone								0.354
	Never/Rarely	247	63.3	107	64.1	140	62.8	
	Sometimes	113	29.0	44	26.3	69	30.9	
	Most of the times/Always	30	7.7	16	9.6	14	6.28	
Alcohol use experimentation								0.106
	No	280	71.8	127	76.1	153	68.6	
	Yes	110	28.2	40	23.9	70	31.4	
Tobacco experimentation								0.794
	No	370	94.9	159	95.2	211	94.6	
	Yes	20	5.1	8	4.8	12	5.4	

a Absolute frequency.

b p value calculated by Pearson's chi-square or Fisher's exact test.

When considering the family and social context, 67.7% of the adolescents lived with both parents. For 62.6% of the adolescents, the parents knew what they did in their free time most of the time or always, 42.3% had their problems and concerns understood by their parents most of the time or always, and 71.1% reported that the parents never or rarely looked into their things. Most adolescents had three or more friends and 63.3% never or rarely felt alone. Tobacco and alcohol use experimentation were reported by 71.8% and 5.1% of the adolescent, respectively (Table 2).

Differences between *quilombola* and *non-quilombola* adolescents were observed for the variables race (p = 0.003), economic level (p < 0.001), and current work (p = 0.023) (Table 2).

The prevalence of sexual intercourse was significantly higher among individuals who worked, did not attend school, did not live with parents, reported that the parents never or rarely understood their problems and concerns, and who sometimes felt alone. Tobacco and alcohol use experimentation showed prevalence of sexual intercourse of 63.6% and 90.0%, respectively (Table 3).

**Table 3:** Prevalence, prevalence ratio, and 95% confidence interval for occurrence of sexual intercourse according to the variables studied. Research ADOLESCER, Bahia, Brazil, 2015.

Variable		Total			*Quilombola*			*Non-quilombola*		
		P(%)*	Crude PR	IC95%	P(%)*	Crude PR	IC95%	P(%)*	Crude PR	IC95%
*Quilombola*										
	No	25.1	1.00	-	-	-	-	-	-	-
	Yes	28.1	1.12	0.80–1.56	-	-	-	-	-	-
Sex										
	Male	28.4	1.00	-	32.9	1.00	-	25.4	1.00	-
	Female	24.5	0.86	0.62–1.20	24.2	0.73	0.45–1.19	24.8	1.04	0.64–1.53
Race										
	Non-black	18.5	1.00	-	33.3	1.00	-	12.3	1.00	-
	Black	28.9	1.56	0.98–2.50	27.1	0.81	0.47–1.48	30.4	2.60	1.24–4.93
Economy level										
	B or C	27.2	1.00	-	32.5	1.00	-	25.2	1.00	-
	D or E	25.9	0.96	0.68–1.37	26.8	0.82	0.48–1.40	25.0	1.00	0.63–1.56
Current work										
	No	18.7	1.00	-	21.1	1.00	-	16.5	1.00	-
	Yes	38.9	2.08	1.50–2.91	43.4	2.06	1.30–3.30	36.5	2.20	1.37–3.54
Currently attends school										
	No	85.7	1.00	-	83.3	1.00	-	88.2	1.00	-
	Yes	20.6	0.25	0.20–0.32	21.5	0.27	0.19–0.37	19.9	0.26	0.20–0.31
Family composition										
	Live with both parents	24.6	1.00	-	27.1	1.00	-	22.9	1.00	-
	Only live with the father or mother	24.2	1.02	0.64–1.50	24.4	0.90	0.48–1.70	24.0	1.08	0.59–1.85
	Do not live with parents	45.7	1.87	1.22–2.82	42.1	1.55	0.84–2.87	50.0	2.18	1.23–3.85
Frequency in which parents knew what they did in their free time										
	Most of the time/Always	23.0	1.00	-	25.5	1.00	-	21.3	1.00	-
	Sometimes	27.1	1.18	0.75–1.85	29.0	1.14	0.60–2.18	25.6	1.21	0.65–2.25
	Never/Rarely	32.9	1.43	1.06–2.14	34.3	1.34	0.78–2.38	31.6	1.48	0.84–2.61
Frequency in which parents understood problems and concerns										
	Most of the time/Always	20.3	1.00	-	22.9	1.00	-	18.3	1.00	-
	Sometimes	27.4	1.35	0.89–2.06	34.0	1.51	0.85–2.63	22.5	1.23	0.68–2.27
	Never/Rarely	32.7	1.61	1.06–2.45	27.9	1.22	0.64–2.33	36.4	2.00	1.14–3.47
Frequency in which parents looked into their things without permission										
	Most of the time/Always	30.2	1.00	–	26.3	1.00	–	32.4	1.00	–
	Sometimes	29.3	0.97	0.55–1.72	30.0	1.14	0.42–3.13	29.0	0.90	0.46–1.80
	Never/Rarely	24.1	0.80	0.50–1.26	27.6	1.05	0.50–2.35	21.10	0.65	0.37–1.16
Close friends										
	< 3	25.8	1.00	–	14.8	1.00	–	33.3	1.00	–
	≥ 3	26.5	1.03	0.66–1.61	30.7	2.07	0.81–5.31	23.4	0.70	0.42–1.17
Felt alone										
	Never/Rarely	23.1	1.00	–	26.2	1.00	–	20.7	1.00	–
	Sometimes	31.9	1.38	1.17–2.00	29.6	1.13	0.65–2.02	33.3	1.61	1.04–2.56
	Most of the times/Always	33.3	1.44	0.86–2.53	37.5	1.43	0.70–2.92	28.6	1.38	0.58–3.36
Alcohol use experimentation										
	No	11.8	1.00	–	14.2	1.00	–	9.8	1.00	–
	Yes	63.6	5.41	3.80–7.68	72.5	5.11	3.20–8.18	58.6	6.00	3.60–10.1
Tobacco experimentation										
	No	23.0	1.00	–	25.2	1.00	–	21.3	1.00	–
	Yes	90.0	3.92	3.09–5.00	87.5	3.48	2.40–5.07	91.7	4.30	3.15–5.87

* P(%): prevalence of sexual intercourse (in percentage).

Among the *quilombola* adolescents, this prevalence was higher among adolescents who worked, did not attend school, and had a tobacco and alcohol use experience. Among the *non-quilombola* adolescents, the occurrence of sexual intercourse was higher when they were black, worked, did not attend school, did not live with parents, when the parents never or rarely understood problems and concerns, felt alone, and had a tobacco and alcohol use experience (Table 3).

The numerical variables age and education were significantly associated with higher frequency of sexual intercourse, with prevalence ratios of 1.51 (95%CI 1.42–1.60) and 1.25 (95%CI 1.19–1.31), respectively. This was also observed for *quilombola* (RP = 1.48, 95%CI 1.36–1.62; RP = 1.29, 95%CI 1.21–1.38) and *non-quilombola* adolescents (RP = 1.53, 95%CI 1.42–1.65; RP = 1.24, 95%CI 1.16–1.32).

Age (PR = 1.42) and alcohol use experimentation (PR = 2.41) were positively associated with sexual intercourse after adjustment. In the *quilombola* stratum, age (RP = 1.37), having three or more close friends (PR = 1.37), and alcohol use experimentation (PR = 2.60) were associated. In the *non-quilombola* stratum, sexual intercourse was independently associated with age (PR = 1.43), black persons (PR = 2.06), and alcohol use experimentation (PR = 2.68) (Table 4).

**Table 4:** Factors associated with sexual intercourse, after multivariate analysis with total sample and place of residence. Research ADOLESCER, Bahia, Brazil, 2015.

Variable		Total		*Quilombola*		*Non-quilombola*	
		RP ajustada	IC95%	RP ajustada	IC95%	RP ajustada	IC95%
Age		1.42	1.33–1.52	1.37	1.25–1.51	1.43	1.38–1.60
Race							
	Non-black	-	-	-	-	1.00	-
	Black	-	-	-	-	2.06	1.08–3.65
Frequency in which parents understood problems and concerns							
	Most of the time/Always	1.00	-	-	-	1.00	-
	Sometimes	1.05	0.78–1.50	-	-	0.77	0.46–1.28
	Never/Rarely	1.31	0.94–1.83	-	-	1.30	0.79–2.07
Close friends							
	< 3	-	-	1.00	-	-	-
	≥ 3	-	-	1.37	> 1.00–4.51		-
Alcohol use experimentation							
	No	1.00	-	1.00	-	1.00	-
	Yes	2.41	1.70–3.46	2.60	1.58–4.07	2.68	1.58–4.60

## DISCUSSION

The results show that the sexual behavior of rural adolescents is similar to those living in urban areas, and individual and family factors and unfavorable behaviors are associated with the experience of sexuality among rural adolescents.

Sexual intercourse in life in rural adolescents (26.4%) was similar to that found in Brazilian studies^5^ ^,^ ^d^, but it was superior to those found in international studies^3^ ^,^ ^6^, both for *quilombola* and *non-quilombola* adolescents. Results of the National Student Health Survey (PeNSE), carried out in 2012 with students aged 13 to 15 years, showed that 28.7% of the interviewees had sexual intercourse^7^. This result was different for the Brazilian Northeast region (24.9%) and for the capital of Bahia (36.5%)^8^. In the city of Goiânia, Brazil, the prevalence of this behavior was 26.5%^5^. These similarities found in distinct populations are possibly related to access to the media, such as radio and TV, or even the Internet. Sexuality, understood as a social and historical construction, has found greater openness in the media for discussions about its experience^9^. This situation may influence the sexual behavior of adolescents regardless of their residence in rural or urban areas.

A study carried out in rural California, with adolescents aged between 12 and 17 years, has found a prevalence of 15.8% of sexual intercourse^3^. In Malaysia, with rural female adolescents, 3.2% of the interviewees had sexual intercourse^2^. The results presented by these studies showed lower prevalence of sexual intercourse among adolescents in relation to those found in this study. However, such a comparison should take into account the cultural differences between the countries where the studies were carried out and that the study population differs regarding the approach of only one sex^2^ or different age groups of adolescents^2^ ^,^ ^3^.

The age of the first sexual intercourse in the rural area was lower than that found by the National Demographic and Health Survey conducted in 2006, in which the average age of sexual initiation of the Brazilian female population was 17.9 years^g^. In 2009, the age of the first sexual intercourse among adolescents interviewed by the PeNSE was predominantly 13 and 14 years (26.1% and 26.5%, respectively). The results of the 2012 PeNSE^d^ kept the highest occurrence of first sexual intercourse in this age group but with a decrease in proportions (13.7% for 13 and 22.9% for 14 years).

Considering only the age of early sexual initiation (adolescents who started their sex life aged 15 years or less)^1^, this study presented a frequency of 60.4%, being slightly higher in *quilombolas* (61.8%) than in *non-quilombolas* (58.7%). There is a consensus in the literature that early sexual initiation is a risky behavior that may increase the chances of multiple sexual partners throughout life^10^ ^,^ ^11^. This situation may favor a greater chance of exposure to some STIs, such as HPV (human papilloma virus)^10^. Moreover, Teixeira et al.^12^ and Aerts et al.^13^ have reported that adolescents who have early sexual initiation may have less access to information such as contraceptive methods and where to get condoms.

The age of first intercourse ranged from six to nineteen years, possibly indicating situations of sexual violence in childhood. The stratification of this variable, according to the place of residence, showed that the earliest ages occurred among the *quilombola* adolescents. This reveals the need for greater vigilance of services that work directly with children and adolescents in the region, such as schools and Family Health Strategy teams, in order to perceive possible signs of sexual abuse and notify social agencies, such as Child Protective Councils and Public Prosecutors.

The use of condom by 77.7% of the adolescents showed a value above the national average of PeNSE^d^ for Brazilian capitals and for the Northeast region (75.3% and 74.0%, respectively). Compared to other studies carried out in Brazil^7^ ^,^ ^14^ ^–^ ^16^ and in Chile^16^, the use of condom in the last sexual intercourse was high for rural adolescents. *Quilombolas* and *non-quilombolas* did not differ in relation to this characteristic.

The 2015 Brazilian HIV/AIDS Epidemiological Bulletin revealed an increase in the incidence of AIDS between the ages of 15 and 19, from 2.1 to 6.7 cases per 100,000 individuals, from 2005 to 2014^h^. The emphasis on the use of condoms as a method of simultaneous protection – contraception and prevention of sexually transmitted infections (STI) – is a necessary proposal among adolescents, considering the important percentage of rural adolescents who were exposed to STI in the last sexual intercourse (23.3%).

Studies have identified an association between older adolescents and sexual initiation^5^ ^,^ ^7^ ^,^ ^17^. Our research corroborates these findings, since the one-year increase in age increased the prevalence of sexual intercourse by 42%, which is similar for both *quilombolas* and *non-quilombolas*. Therefore, this is an important factor to be considered in the development of actions to promote the sexual health of rural adolescents. We emphasize that this study included adolescents who were not included in the regular educational system and those who studied at night, which allowed a better characterization of the diversity of conditions of the studied population.

The frequency with which parents understood the problems and concerns of the adolescents showed to be an important variable to explain the final regression model for the total sample and for the *non-quilombola* stratum. As the first space of interaction of the adolescent, the family can participate more actively in the transformations experienced by adolescents and help them address worries and doubts^8^. A distancing from the family and, consequently, a greater search for peers, can influence the sexual behavior of adolescents^17^. Among the *quilombola* adolescents, the higher number of close friends increased the prevalence of sexual intercourse by 37%.

Among adolescents, those who had an alcohol use experience had 1.41 times more prevalence of sexual intercourse. Among the *quilombola* and *non-quilombola* adolescents, alcohol use experimentation increased this prevalence by 1.60 and 1.68 times, respectively. Studies have indicated an association between the use of psychoactive substances and the report of sexual intercourse^5^ ^,^ ^7^. The effects of alcohol on the human body cause disinhibition and may encourage individuals to engage in behaviors that they would not do so if sober^16^. Among adolescents, in a municipality within Minas Gerais, there was a greater occurrence of sexual intercourse among individuals who had consumed alcohol (43.8% had sexual intercourse under the influence of alcohol and 21.4% of them did not always use a condom)^16^. This scenario exposes the need for sexual education combined with preventive approaches in relation to alcohol use, in addition to facilitating the access to condoms^7^.

For rural adolescents, school, as an important space for socialization during adolescence, was the main source of guidance on pregnancy (53.9%), AIDS and other STI (72.3%), and how to acquire free condoms (46, 4%), for both *quilombolas* and *non-quilombolas*. We can perceive the need to qualify educators and strengthen the intersectoral partnerships between education and health to make the classroom a space for discussions about sexual health in adolescence.

This study presents some limitations. Because this is a cross-sectional study, we cannot infer the temporal nature of some of the associations observed. In addition, the fact that the questionnaires were applied by an interviewer suggests the possibility of information bias because, for example, of the embarrassment related to some questions. To avoid this problem, in addition to the training, interviews were conducted in an isolated place at home, with interviewers of the same sex and age group close to the adolescents (young).

## CONCLUSION

When we investigate the sexual behavior of rural adolescents, we perceive that there are situations that weaken the autonomous and healthy exercise of sexuality in this population. Lack of information and exposure to unfavorable behaviors, such as alcohol use, are adverse conditions that need to be addressed in health promotion strategies and strategies for the prevention of diseases.

The sexual and reproductive health care of adolescents comprises a set of actions that have a privileged locus in the primary health care. Its proximity to families and the better knowledge of the culture and vulnerabilities of the territory make it possible to develop more effective actions for the population. In the rural area, where there is greater difficulty in accessing services, the health sector needs to act more actively in partnership with the education sector, to enhance the spaces already available and to develop strategies for creating new care spaces, aimed at reaching adolescents who do not attend school.

## Footnotes

a Instituto Brasileiro de Geografia e Estatística. Censo Demográfico 2010: População geral distribuída por idade, sexo e local de residência. Rio de Janeiro: IBGE; 2010 [cited 2015 May 25]. Available from: http://www.censo2010.ibge.gov.br/sinopse/index.php?dados=12

b Ministério da Saúde (BR), Secretaria de Gestão Estratégica e Participativa, Departamento de Apoio à Gestão Participativa. Política Nacional de Saúde Integral das Populações do Campo e da Floresta. Brasília (DF): Editora do Ministério da Saúde; 2013 [cited 2015 Jun 18]. Available from: http://bvsms.saude.gov.br/bvs/publicacoes/politica_nacional_saude_populacoes_campo.pdf

c Ministério da Saúde (BR), Secretaria de Atenção em Saúde, Departamento de Ações Programáticas Estratégicas. Diretrizes nacionais para a atenção integral à saúde de adolescentes e jovens na promoção, proteção e recuperação da saúde. Brasília (DF); 2010 [cited 2015 Jun 15]. (Série A. Normas e Manuais Técnicos). Available from: http://bvsms.saude.gov.br/bvs/publicacoes/diretrizes_nacionais_atencao_saude_adolescentes_jovens_promocao_saude.pdf

d Instituto Brasileiro de Geografia e Estatística. Pesquisa Nacional de Saúde do Escolar 2012. Rio de Janeiro: IBGE; 2013 [cited 2015 May 15]. Available from: http://biblioteca.ibge.gov.br/visualizacao/livros/liv64436.pdf

e Instituto Brasileiro de Geografia e Estatística. Pesquisa Nacional de Saúde 2013: percepção do estado de saúde, estilos de vida e doenças crônicas: Brasil, grandes regiões e unidades da federação. Rio de Janeiro: IBGE; 2014 [cited 2015 May 10]. Available from: ftp://ftp.ibge.gov.br/PNS/2013/pns2013.pdf

f Associação Brasileira de Empresas de Pesquisa. Critério de classificação econômica Brasil 2014 (Base LSE 2012). São Paulo: ABEP; 2014 [cited 2015 May 14]. Available from: http://www.abep.org/criterio-brasil

g Ministério da Saúde (BR). Pesquisa Nacional de Demografia e Saúde da Criança e da Mulher (PNDS-2006). Brasília (DF); 2009 [cited 2015 Jun 26]. Available from: http://bvsms.saude.gov.br/bvs/publicacoes/pnds_crianca_mulher.pdf

h Boletim Epidemiológico HIV. Brasília (DF): Ministério da Saúde; 2015;4(1) [cited 2017 Jul 14]. Available from: http://www.aids.gov.br/pt-br/pub/2015/boletim-epidemiologico-hivaids-2015
